# Assessing the association between elements of health inequalities and the risk of bacterial infection in high-income countries: a systematic scoping review

**DOI:** 10.1099/acmi.0.000956.v4

**Published:** 2026-06-22

**Authors:** Nketia Obed-Arthur, Ella Casale, Eleanor J. Harvey, Caroline De Brún, Viviana Finistrella, Ellie Gilham, Diane Ashiru-Oredope

**Affiliations:** 1 Pharmacy Department, Ashford and St. Peter’s NHS Hospitals Foundation Trust, Chertsey, Surrey, UK; 2 AMR and HCAI, Division, United Kingdom Health Security Agency (UKHSA), London, UK; 3 School of Pharmacy, University College London, London, UK

**Keywords:** antibiotic resistance, antimicrobial stewardship, disparities, equity, inequality, inequity

## Abstract

**Introduction.** There is sufficient evidence to support the notion that the rate of infection is higher in vulnerable groups than in the wider population. This scoping review sought to further elucidate the relationship between health inequalities and the risk of bacterial infections in high-income countries.

**Methods.** Factors associated with health inequalities were defined as socioeconomic status and deprivation (employment status, income levels and deprivation categories), protected characteristics (age, gender, ethnicity, sexual orientation and disability), inclusion health groups (migration status, sex workers, people who inject drugs and the homeless) and geography (urban or rural dwelling). The published literature search was conducted on Embase, Google and Medline with a grey literature search also conducted. Exclusion criteria were defined as papers that were either published pre-2010, set in low- or middle-income countries or that covered viral, parasitic or fungal pathogens. Key pathogens and infections to include were determined by review of the UK antimicrobial resistance National Action Plan.

**Results.** The search yielded 343 papers, of which 70 were eligible for review. The most frequently reported infections were sexually transmitted infections, infectious diseases, e.g. *Mycobacterium tuberculosis*, and sexually transmitted blood-borne infections. Most papers reviewed cited multiple health inequalities, most notably ethnicity (21/70), race (18/70), age (14/70), sex (13/70) and socioeconomic status and deprivation and inclusion health groups (sex workers and people experiencing drug and alcohol dependence).

**Conclusion.** Evidence has emerged highlighting an important association between factors commonly associated with health inequalities and the increased risk of bacterial infections within high-income countries. Notably, ethnic populations, members of the LGBTQ+ community, people experiencing homelessness and those of a poor socioeconomic status are at a higher risk of bacterial infection.

**Impact Statement**

This scoping review provides a comprehensive analysis of the impact of socioeconomic status, deprivation, protected characteristics, inclusion health groups and geography on the risk of bacterial infection in high-income countries from 2010 to 2021. By focusing on these factors, the review addresses a gap in the literature, which has predominantly concentrated on low- and middle-income countries. The findings highlight how vulnerable populations in affluent societies face disproportionate risks of bacterial infections, emphasizing the multifaceted nature of health inequalities, even in economically developed regions.

By expanding the scope of research to include high-income settings, this review adds to the literature by providing valuable insights into the pervasive nature of health disparities and the need for inclusive strategies in infection management and prevention across diverse populations. It underscores the importance of considering a broader range of determinants beyond clinical and biological factors when addressing infection risks. The review also calls attention to the necessity for tailored public health interventions that address these inequities, thereby contributing to more equitable healthcare outcomes. This scoping review also provides an important state of the literature prior to the increased focus on health inequalities in infections within nations following the coronavirus SARS-CoV-2 pandemic.

## Data Summary

All data associated with this work is reported within the article.

## Introduction

The burden of infectious disease disproportionately impacts the vulnerable in society and there is evidence of increased risk of infection in these groups [1,5]. Whilst clinicians are often supported by clinical guidelines, the diagnosis of infection and decision-making practices focused on infection management can prove challenging. Treatment decisions are likely to be influenced by other factors, such as antimicrobial resistance (AMR) and the nature of the patient population that the antimicrobials are being used in [6,7]. Other factors, such as cultural behaviours, socioeconomic deprivation and migration status, also add to the complexity of managing infection effectively [8,9]. A retrospective study conducted by Donnelly and colleagues found that those residing in more socioeconomically affluent areas had a lower risk of hospitalization for sepsis [10]. Similar evidence has also demonstrated that low income and unemployment are predictive factors for invasive bacterial infection [11].

In less economically developed countries, there is a relationship between factors associated with health inequalities and the risk of bacterial infection. A cohort study conducted in Kenya found that children aged five and below who were malnourished or dehydrated were at a greater risk of carrying *Campylobacter* infections and subsequent mortality [12]. Evidently, there is well-recognized impact of inequalities within low–middle-income countries and the need to combat pathogenic bacteria, through prevention or better treatment. Conversely, to the best of our knowledge, when commencing this review, no others had been published that assessed the association between health inequalities and the risk of bacterial infection in high-income countries.

In this scoping review, we examine evidence from 2010 to 2021 to investigate the impact of socioeconomic status, deprivation, protected characteristics, inclusion health groups and geography on the risk of bacterial infection in high-income countries.

## Methods

This review was informed by the protocol published by Page *et al. a*nd followed the Preferred Reporting Items for Systematic Reviews and Meta-Analyses (PRISMA) statement (see Material S1, available in the online Supplementary Material) [13].

### Research question

The proposed research question was as follows: What evidence is there for an association between factors commonly associated with health inequalities and the risk of bacterial infection in high-income countries?

Factors commonly associated with health inequalities were defined as socioeconomic status and deprivation (employment status, income levels and deprivation categories), protected characteristics, as outlined by the UK’s Equality Act 2010 (age, disability, gender reassignment, marriage and civil partnership, pregnancy and maternity, race/ethnicity, religion or belief, sex, sexual orientation), vulnerable groups [migration status, sex workers, people who inject drugs (PWIDs) and the homeless] and geography (urban or rural dwelling).

### Information sources and search strategies

We carried out a scoping review using the search terms in Material S2. The search included literature from 1 January 2010 to 29 April 2021. The search was limited to literature in the English language and the publication types included were primary and secondary research. A Knowledge and Evidence Specialist (author – C.D.) searched Embase, Google Scholar and Medline. The words ‘coronavirus SARS-CoV-2 (COVID-19)’ and ‘pandemic’ were excluded. An initial screen of results was undertaken to remove irrelevant or duplicate literature. Grey literature search was also conducted to identify relevant grey literature published between 1 January 2010 and 2021.

### Selection criteria

Following an initial screening by a Knowledge and Evidence Specialist, the results were screened. Two-weekly co-author meetings between co-authors D.A.O, E.C., E.J.H. and V.F. were conducted virtually on Microsoft Teams to discuss screening and data extraction. Second checks of articles for inclusion or exclusion were performed by two researchers (D.A.O. and V.F.) during virtual meetings; any concerns on specific articles were raised, discussed and resolved (Table 1). Literature that met any of the exclusion criteria was not included. This review specifically focused on bacterial infection, with specific pathogens and infections. Throughout this manuscript, ‘infection’ refers specifically to bacterial infection unless otherwise stated.

**Table 1.** Inclusion and exclusion criteria

Field	Inclusion criteria	Exclusion criteria
Reference type	Journal article	Reviews
Year	January 2010–April 2021	Pre-2010
Pathogen/infection focus	STI (sexually transmitted infections secondary to bacterium) (multiple), *Mycobacterium tuberculosis*, Chlamydia, infectious disease, *Clostridium difficile*, *Helicobacter pylori*, rhinosinusitis, sepsis, frequent ear infection (FEI), gingivitis, pneumonia, Shiga toxin-producing *Escherichia coli*, RTIs, *Staphylococcus*, MRSA/MSSA, skin infection, *Mycoplasma genitalium*, foodborne pathogens, *Staphylococcus aureus*, syphilis, BSI, *Neisseria, meningitides*, pelvic inflammatory disease	HIV, HPV, hepatitis C, hepatitis B, hepatitis, influenza, Chagas disease, herpes simplex virus type 2, *Trichomonas vaginalis*, HIV/AIDS, HIV/HCV, Epstein–Barr virus, *Pneumocystis jirovecii*, toxocariasis, herpes simplex virus, herpes simplex virus type 1 and 2, rhinovirus/HIV/hepatitis, strongyloidiasis and schistosomiasis, Zika virus, *Giardia lamblia*, pertussis, helminth, chickenpox, cytomegalovirus, HIV, chronic fatigue syndrome, blood-borne viruses, coccidioidomycosis, viral gastrointestinal infections, HIV/syphilis, babesiosis, appendicitis
Setting	Hospital, community	None excluded
Population	Humans	Animals
Sample size	n/a	None excluded
Health inequality	**Socioeconomic status and deprivation:** employment status, income level, deprivation category **Protected characteristics (Equality Act 2010):** age, disability, gender reassignment, marriage and civil partnership, pregnancy and maternity, race (ethnicity), religion or belief, sex, sexual orientation **Vulnerable group:** migration status, homelessness, sex worker, PWIDs **Geography**: urban or rural dwelling	None excluded
Country	High-income countries	Low- and middle-income countries as defined by the Organisation for Economic Co-operation and Development (OECD)

AIDS, autoimmune deficiency syndrome; BSI, bloodstream infection; FEI, frequent ear infection; HIV, human immunodeficiency virus; HPV, human papillomavirus; MRSA, methicillin-resistant *Staphylococcus aureus*; MSSA, methicillin-sensitive *Staphylococcus aureus*; RTIs, respiratory tract infections.

### Data extraction

A data extraction table was developed in Microsoft Excel and included 17 fields: reference type, author, year of publication, title, journal, pathogen, key findings, setting, population, elements of health inequalities covered, country, UK region, exclude/include, keywords, abstract, URL and database.

## Results

**Fig. 1.**
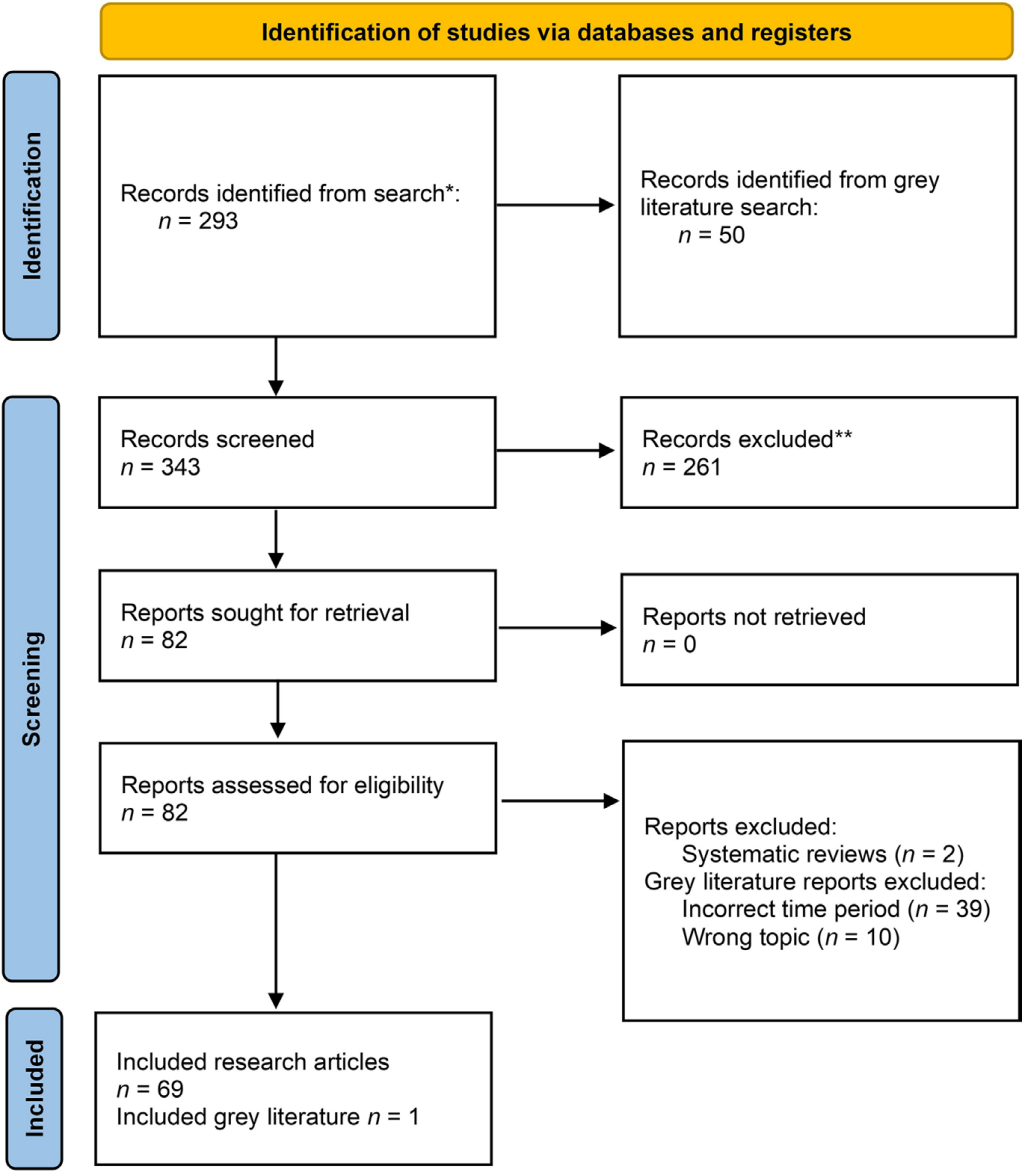
PRISMA flow diagram.

The initial search returned 343 results. A first screen was undertaken excluding 221 results. The remaining 72 results were screened to determine which results addressed the relationship between health inequalities and the risk of bacterial infections within high-income countries with two further papers excluded. These 70 papers were included in the review (Fig. 1). The grey literature search returned 50 results, of which 1 was eligible for inclusion in the review. The eligible article provided a short reference to disparities evident in the epidemiology of sexually transmitted infections (STIs) with populations with factors associated with health inequalities such as age, race and ethnicity, sexual orientation and gender identity, highlighted as being disproportionately affected. However, the primary focus of the article was on identifying interventions to reduce STI incidence rather than to identify the increased risk of infection these populations face.

**Fig. 2.**
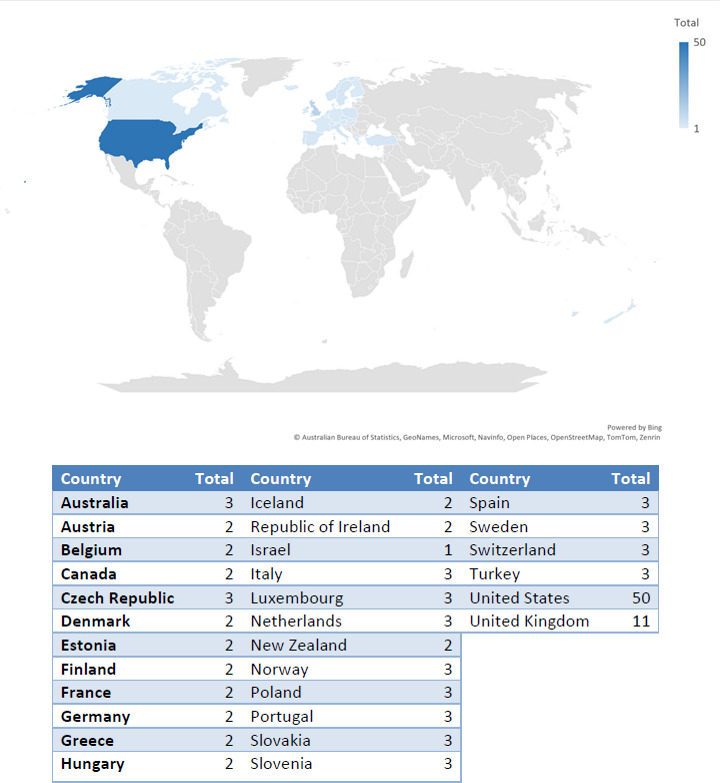
Number of studies published within high-income countries that assess factors commonly associated with health inequalities and risk of bacterial infection.

Included studies spanned 30 countries, with most studies conducted in the USA (Fig. 2). Studies were set within community (60/70) or hospital settings (10/70). Furthermore, ethnicity (21/70), race (18/70), age (14/70), sex (13/70) and socioeconomic status (13/70) were the most studied; see Table 2 for a summary of results.

**Table 2.** Summary of results per factor commonly known to be associated with health inequalities

Factor commonly known to be associated with health inequalities (HI factor)	No. of papers that included HI factor	No. of papers that associated HI factors with infection risk	Summary of results
**Socioeconomic status**	13	8	STI: impoverished men who have sex with men had significantly higher rates of syphilis [14]; poverty and education levels were also associated with higher risk of STI [109] and chlamydia infection [61]
			Tuberculosis: significant association between SES and TB incidence [11,19]; however, patients living in more deprived areas did not appear to have higher probabilities of more advanced disease [20]
			Foodborne pathogens: only one study showed higher incidence of Shiga toxin-producing *E. coli* in lower SES groups with higher odds of hospitalization also seen [24]; others found lower social classes were less likely to contract food or water borne pathogens [11,23]
			Infectious disease: patients with *Neisseria meningitidis* invasive bacterial diseases and blood-borne infectious disease were more likely to live in high levels of neighbourhood poverty [21,110], be unemployed and have a lower educational attainment level
**Income levels**	4	4	TB: income inequality was found to be a strong predictor for TB incidence [18]
			STI: lower income, amongst other factors, was associated with an increased risk of STIs in female young adults and adolescents; STI diagnosis was found to be independently associated with low income; STI risk increased as income level declined [58]
			Ear infection: income below the poverty level predicted incidence of frequent ear infection [22]
**Geography**	3	2	STI: weak evidence that moving to an area with more equitable sex ratios reduces the risk of STI [17]; increased risk of STI also seen within male adolescents and young adults who had moved home four or more times since kindergarten [28]
**Sex workers**	3	3	STI: female sex workers found to have higher risk of some STIs, including gonorrhoea and chlamydia compared to other females [25]; male sex workers also found to have a higher risk of STIs, including HIV, gonorrhoea and chlamydia and reinfection compared to other males [26]; risk of STI in sex workers increased in those who had no contact with an outreach worker, had a non-paying sexual partner or were aged 23–26 years compared to women aged under 22 years [71]
**People experiencing homelessness**	4	3	Rhinosinusitis: high prevalence of potential chronic rhinosinusitis symptoms in the urban homeless population [30]
			TB: groups at greatest risk of TB include those experiencing homelessness [29]; people experiencing homelessness are more frequently seen for head and neck tuberculosis [111]
**Migrants**	10	8	MSSA: immigrants were significantly more likely to present with SSTIs caused by MSSA than US-born patients [112]
			*Helicobacter pylori*: prevalence has declined in the Canadian population but remains high in Indigenous people and recent immigrants [34]
			TB: multiple studies showed TB cases were more likely to be foreign-born [50], including both smear-negative [33] and latent TB cases [35,37, 48, 113]
**Prisoners**	2	2	STI: high prevalence of chlamydia (10.3%) seen in females within a juvenile correction unit [31]
			TB: smear-negative TB cases are more likely to be incarcerated amongst other risk factors [33]
**Sexual orientation and gender identity**	4	2	Non-binary gender identity associated with higher risk of STI; trans females assigned male sex at birth also had higher odds of STI compared to trans males assigned female sex at birth [32]; high prevalence of STIs within older gay and bisexual men; being HIV-positive, condomless receptive sexual activity and intravenous drug use were associated with an increased risk of STI [38]
**Race**	18	18	Chlamydia: high level of prevalence seen within non-Hispanic Black individuals [31,53, 54]; South Asian ethnicity was associated with a lower risk of infection [15]
			STI: in Black females, education did not have the same protective effect against STI that it did within White females [16]; a higher risk of infection was also seen in Black compared to White males [109]
			CDI: White race associated with higher risk of CDI [45]
			FEI: a lower odds ratio for FEI was seen in Black, Hispanic and other race/ethnic groups compared to White children [22]
			Gingivitis: gingivitis was significantly more common in African Americans than other races, except for Native Americans [51],
			Infectious disease: Black maternal race was associated with increased infectious disease death amongst low-birth-weight infants [56]
			*Staphylococcus*: staphylococcal scalded skin syndrome was significantly associated with race, with Black and Hispanic races having a reduced risk and Asian/Pacific Islanders having an increased risk compared to White individuals [55]
			TB: high prevalence and increased risk of TB was consistently seen in non-Hispanic Black and Hispanic populations [35,48, 113]; a high prevalence of TB was also seen in minority race and ethnic groups [29,50, 111]; Hispanic and non-Hispanic people were also found to be less likely to have smear-negative TB [33]
**Ethnicity**	21	18	Chlamydia: high levels of racial disparity in infection rate despite reductions in prevalence within the USA [41]; strong association between race and chlamydia status [70]; infection risks were higher for Black, American Indian/Alaskan Natives, Asian/Pacific Islander and Hispanic women compared to White women after adjusting for individual factors [42]
			STI: independently associated with racial/ethnic identity with a higher incidence of infection seen within Black Caribbean ethnic groups [40]
			CDI: higher incidence rates seen in White compared to Black ethnic groups [44,46]
			*Helicobacter pylori*: despite decreasing prevalence in Western countries, prevalence remains high in migrant communities; African American ethnic groups were also associated with higher odds of *H. pylori* infection [43]
			PID: higher prevalence of PID seen in Black compared to White women if no previous STI had been diagnosed [114]
			RTI: higher risk ratio of RTI in Pakistani men and South Asian women; Pakistani men and women also had higher rates of RTI hospitalization [115]
			Sepsis: a lower prevalence was found in Black compared to White ethnic groups [116]
			Skin infection: high prevalence of infection seen in ethnic groups [117]
			*Staphylococcus aureus*: higher risk of MRSA infection seen in Black and Native American ethnic groups [118]
			TB: Black individuals were more likely to report TB infection compared to White individuals [49]; local transmission was also found to be higher amongst US-born and non-Hispanic Black cases [69]
**Age**	14	8	CDI: older age was independently associated with higher risk of CDI [45]
			Chlamydia: higher prevalence [54,119] and risk [15,53] found within younger age groups
			Gingivitis: significant correlation seen between age and gingivitis [51]
			Infectious disease: children aged 10 years and younger had an increased odds of developing staphylococcus scalded skin syndrome [55]
			TB: in US-born cases, the highest TB prevalence was seen in those aged 65 and over; in non-US-born cases, the highest prevalence was in those aged 45–65 years [113]
**Sex**	13	10	Chlamydia: prevalence found to be higher in females than males [15,54], although one study showed no difference in prevalence by gender [53]
			Gingivitis: significantly higher in males than females [51]
			Infectious diseases: male sex was associated with infectious disease death within both low and normal birthweight infants [55]
			Staphylococcus: females had higher odds of staphylococcus scalded skin syndrome [55]
			STI: gender differences evident in risk factors for STI [28,109]
			TB: older males found to be at higher risk of TB [50]; males were also found to be at greatest risk for recent TB transmission [29]
**Education level**	2	2	Chlamydia: after adjusting for other measures of disadvantage, lower maternal and participant education levels were the strongest risk factors for infection [61]
			STI: individuals with higher education levels had a lower risk of STI infection; however, this relationship was not seen in Black females [16]
**Pregnant women**	1	1	STI: STI prevalence amongst pregnant women was influenced by age, ethnicity, unmarried status, education level, income level and possession of pre-pregnancy health insurance [57]

CDI, *Clostridium difficile*; FEI, frequent ear infection; HIV, human immunodeficiency virus; MSSA, methicillin-sensitive *Staphylococcus aureus*; PID, Pelvic inflammatory disease.

The most frequent bacterial infections that were cited were STIs (15/70) and *Mycobacterium tuberculosis* (TB) (15/70) (Table 3).

**Table 3.** The number of infections investigated by papers included within this review

Pathogen/infection	No. of papers
STI (multiple)	15
*M. tuberculosis*	15
Chlamydia	10
Infectious disease (multiple)	3
*Clostridium difficile*	4
*Helicobacter pylori*	3
*Staphylococcus*	3
Rhinosinusitis	2
Sepsis	2
Bloodstream infection	1
Foodborne pathogens	1
Frequent ear infection (FEI)	1
Gingivitis	1
*Mycoplasma genitalium*	1
*Neisseria meningitidis*	1
Pelvic inflammatory disease	1
Pneumonia	1
Respiratory tract infection	1
Shiga toxin-producing *Escherichia coli* (STEC)	1
Skin infection	1
Syphilis	1
**Total**	**69**

### Socioeconomic characteristics (income, education, geography and deprivation)

Our search reported a correlation between various socioeconomic characteristics and the risk of bacterial infection.

#### Education

Our search found a negative correlation between education level and incidence of STIs with university education associated with a lower risk of STIs [14,16]; however, the protective effect of education appeared to differ by racial group. Black females with college-level education had significantly higher predicted probabilities of having an STI (12.4% self-reported; 13.4% assay-diagnosed) compared with White females who had less than a high school diploma [14,16]. This suggests that whilst education may be protective within racial groups, racial disparities in STI risk persist and may be larger than the protective effect of education alone.

#### Geography

Our results found no significant correlation between risk of infection and geographical location. Cooper and colleagues reported that individuals in the USA who relocated from public housing complexes had a lower incidence of STIs; however, this was due to more equitable sex ratios rather than any other environmental condition [17].

#### Deprivation

Our results also found a negative relationship between socioeconomic status and the incidence of TB cases in Europe [18]. Similar trends were identified in reports conducted in the USA, with evidence demonstrating that there were greater incidences of TB in socioeconomically deprived areas than in more affluent areas [19,20]. Furthermore, in the USA, those with invasive bacterial diseases, blood-borne infectious diseases, TB and antibiotic-resistant infections were statistically more likely to be unemployed, attained fewer educational qualifications and have lower incomes [11,21]. Frequent ear infections in children were also reported to be associated with incomes below the standard poverty level in the USA [22]. Shiga toxin-producing *E. coli* (STEC) infection was more prevalent in high than low socioeconomic classes in Connecticut, the USA [23] and the UK [24], whereas non-STEC infection was more prevalent in low socioeconomic classes.

### Inclusion health groups

#### Sex workers

The evidence suggested a strong relationship between sex work and the risk of bacterial STIs. Both male and female sex workers were at a statistically significantly greater risk of chlamydia and gonorrhoea than their non-sex worker counterparts [25,26].

Platt and colleagues found that the risk of STIs in sex workers increased in those who had no contact with an outreach worker, had a non-paying sexual partner or were aged 23–26 years compared to women aged under the age of 22 [27]. This substratum of female sex workers also reported lower levels of contraceptive use, which partially explains the increased exposure to chlamydia and syphilis. There was no difference in the incidence of STIs between migrant and non-migrant female sex workers.

Furthermore, young people in lower socioeconomic strata who engaged in exchanging sexual intercourse for financial support had an increased risk of STI [28]. Community-level economic factors appeared relevant, as those who disagreed with the notion that young people in their community exchanged sex for money had a lower likelihood of having an STI [28].

#### People experiencing homelessness

Homelessness was associated with an increased risk of infection, notably tuberculosis [29]. Chronic sinusoidal symptoms were more prevalent in longer durations of homelessness, and this was associated with poorer quality of life [30].

#### People who inject drugs

Evidence from the broader literature suggests that PWIDs are at increased risk for bacterial infections, including skin and soft tissue infections and bloodstream infections associated with injection practices. However, our search did not return studies specifically examining bacterial infection risk in the population that met our inclusion criteria.

#### Prisoners

Chlamydia prevalence was high amongst incarcerated populations, namely including female entrants to juvenile prisons [31]. Torrone and colleagues found that extensive screening in prisons is feasible but also offers a more accurate picture of chlamydia prevalence in incarcerated individuals [32]. Smear-negative TB cases were also more likely amongst incarcerated individuals [33].

#### Migrants

Evidence suggests that migrant status is associated with infectious disease risk. In Canada, despite a decreasing occurrence of *Helicobacter pylori* within the Canadian population, cases of the pathogen remained high amongst immigrants and ‘indigenous people’ [34].

Similar trends applied to the risk of tuberculosis. In the USA, foreign-born individuals constitute 73% of the total number of cases which is disproportionately higher than those from US-born cases [29,35]. Between 2007 and 2011, a decrease in the number of migrants from Mexico to the USA was associated with a decrease in the number of TB cases in the overall population [36]. Dale *et al*. reported that the proportion of Australian residents with tuberculosis increased from 4.6% in 2006 to 5.1% in 2016, due to an increase in immigration to the country [37]. Of all residents estimated to have TB in 2016, 93.2% were foreign-born.

### Protected characteristics

#### Sexual orientation and gender identity

Casual sexual activity between gay and bisexual individuals was associated with an increased risk of chlamydia and gonorrhoea [38]. Leichliter *et al*. suggested that policies relating to sexual orientation, such as marriage, housing and hate crimes, had a strong correlation with syphilis in this specific population [14].

The odds of bacterial STIs were 4.06 times greater for trans female youth assigned a male sex at birth compared to trans male youth assigned a female sex at birth [39]. Those who identified as nonbinary were also at higher risk of an STI compared to those who identified with their gender assigned at birth. No identified studies in our search made direct comparisons between the incidence of STIs in heterosexual and non-heterosexual cohorts.

#### Race and ethnicity

Our search results found that in the UK, incidental STI diagnoses were more common in heterosexual individuals from the Afro-Caribbean community when compared to their White British counterparts [40]. Tian *et al*. reported that the racial disparity of the incidence of infection for chlamydia was 8.1% for women and 9.0% for men when comparing socioeconomically disadvantaged Black individuals to socioeconomically disadvantaged White individuals in the USA [41]; other studies reported similar findings [42]. Furthermore, Black females had significantly higher predicted probabilities of having an STI compared with White females in the USA [16].

Regarding *H. pylori*, African American populations had increased odds of positive serology of CagA-positive strains compared to White American populations, even after controlling for socioeconomic status [43].

Our findings also found higher cases of *Clostridium difficile* in White patients than in non-White patients (Black, Hispanic, Asian and Native American) [44,46]. Whilst White ethnicity was associated with a higher incidence of *C. difficile*, Black patients had statistically higher rates of severe *C. difficile* and *C. difficile*-associated mortality [46]. A similar trend was found regarding *H. pylori*; in the Netherlands, the prevalence of *H. pylori* in Dutch women (24%) was significantly lower than the prevalence of *H. pylori* in non-Dutch women [47].

With regard to other infectious diseases, Black individuals were more likely to report tuberculosis disease, after controlling for socioeconomic factors [48,49].

#### Age

Age was associated with increased infection prevalence. In elderly populations, an increased incidence of *M. tuberculosis*, *C. difficile* and gingivitis were reported [50,52]. Conversely, STIs were more prevalent in younger populations; Beydoun and colleagues reported that the prevalence of chlamydia was higher in people younger than 25 compared to those over the age of 25 [53]. Younger age groups in paediatric populations were at greater risk of more severe cases of pneumonia and other infectious diseases such as bacteraemia and septicaemia.

#### Sex

Results for associations between sex and infection risk were dependent on the type of infection. In terms of STIs, female sex was correlated with a higher incidence of STIs than male sex. Datta and colleagues conducted a population-based assessment of national trends in chlamydia prevalence in the USA; the assessment found that prevalence was higher amongst females than males [54]. In the same study 10 years later, there was an estimated 40% reduction in prevalence amongst male participants aged 14 to 39 years, but no change in prevalence amongst females aged 14 to 25 years. In terms of infectious diseases, Moonan *et al*. reported a higher prevalence of *M. tuberculosis* in male compared to female populations [29].

In paediatric populations, Staiman and colleagues reported that incidences of staphylococcal scalded skin syndrome were significantly associated with female sex [55], whereas Person *et al*. reported that male sex was associated with higher infectious disease mortality within both low and normal birthweight infant populations [56].

#### Pregnancy and maternity

Williams and colleagues reported that pregnant women had a higher incidence of STIs than non-pregnant women [57]. Amongst pregnant women, the prevalence of STIs was higher amongst younger women, women of Black race/ethnicity, unmarried women, women with no college education, women with low income and women with no pre-pregnancy health insurance.

## Discussion

To the best of our knowledge, this is the first comprehensive scoping review assessing evidence on the association of factors commonly associated with health inequalities and the risk of bacterial infection in high-income countries. Ethnicity/race, age and sex were the most frequently assessed factors, followed by socioeconomic status. Overall, the evidence showed that certain racial/ethnic groups, migrants, those who are homeless, those of a poor socioeconomic status, non-heterosexual groups and those involved in sex work were at higher risk of infection.

### Socioeconomic characteristics (income, education, geography and deprivation)

Those with low income appeared to be at a greater risk of infection. With regard to STIs, Boyer’s work suggested that young people in lower socioeconomic strata may feel compelled to exchange sex for financial gain to support themselves. This is echoed by Harling *et al*., who also proposed a similar behaviour of using sex for economic purposes [58]. Hogben *et al*. proposed that sex is used as a psychosocial coping mechanism for the stresses associated with low income [59], which may suggest that the high incidence of STIs could be symptomatic of low income itself. This is supported by Ibragimov’s findings, which suggest that between 2003 and 2015, states within the USA with higher minimum wages had lower frequencies of STIs [60].

Lower levels of education were also associated with greater risks of STIs. Williams *et al*. and Crichton *et al*. reported a greater risk of chlamydia in young people with lower maternal educational attainment and lower educational attainment [57,61]. Education offers individuals essential information surrounding safe sexual practices and the importance of regular health check-ups. Informed individuals may be more likely to make responsible decisions regarding their sexual health, which may lower the incidence of risky sexual behaviours, therefore lowering the incidence of STIs. Preventative interventions, including health education programmes and targeted diagnostic testing strategies, may reduce the incidence of STIs; this is supported by the observation that targeted interventions commissioned by local authorities in the UK were more likely to be attained in areas of higher socioeconomic deprivation [62].

Our results also demonstrated a positive correlation between deprivation and risk of infection. Tuberculosis is a poverty-driven infection, with evidence supporting the notion that the incidence of *M. tuberculosis* is proportional to socioeconomic deprivation [63,65]. Impoverished communities, overcrowded living conditions and limited access to sanitation may create environments conducive to the transmission of *M. tuberculosis* which is often spread through droplet vectors [66,68]. This is supported by a study conducted by Noppert *et al*., who proposed that in the USA, geographic areas with a high proportion of TB cases were likely due to ongoing transmission between patients, rather than secondary to reactivated cases of latent TB [69].

No evidence supporting a link between geographic location and increased risk of infection was found.

### Inclusion health groups (sex workers, people experiencing homelessness and migrants)

Our findings reported that sex workers are at a greater risk of STIs than non-sex workers. Sex workers face heightened levels of criminalization and social stigma [70], which may discourage them from coming forward for sexual health screening out of fear of judgement or incarceration. Fear of arrest and criminal prosecution may also pressure sex workers to rush negotiations with service users, resulting in a reduction in client screening of potential infection or work in unsafe, isolated locations to avoid police detection. This notion is supported by the observation that sex workers who did not face repressive policing were 30% more likely to screen clients for STIs and engage in safer sexual practices [71].

Homelessness was also associated with increased risk of infection. An Australian study reported that *Mycoplasma genitalium* infection was prevalent in marginalized young people in Melbourne, Australia [68]; most notably, a greater proportion of homeless women were at risk of STIs compared to homeless men. Other evidence supports this observation, with Williams and Bryan reporting a greater prevalence of *chlamydia* and *gonorrhoea* in homeless adult women compared to homeless adult men [72].

Migrant status was also associated with a greater risk of infections, such as methicillin-resistant *Staphylococcus aureus* and tuberculosis. This could be explained by evidence detailing that migrants experience a higher risk of infection due to a lack of access to healthcare systems, either due to legal barriers or an inability to afford necessary healthcare [73,77]. This lack of access to treatment may also explain the greater prevalence of tuberculosis in migrant populations. Public health campaigns could provide resources to ensure migrant communities have access to appropriate infection prevention methods, such as vaccination.

Prisoners were reported to have higher incidences of chlamydia [31]; some evidence reports that this is due to dense population, poor living conditions and high-risk sexual behaviours [77,79]. These behaviours have been reported to correlate with low educational levels, which could imply that prison systems should invest in wider screening and education systems to reduce the incidence of infection in inmates [80].

### Protected characteristic groups (sexual orientation and gender identity, race/ethnicity, age, sex, pregnancy and maternity)

Analysis of the relationship between sexual orientation and the risk of infection is complex. Our search returned no results which directly compared the prevalence of infection in heterosexual populations to non-heterosexual populations. It would be important to understand what specific risk factors or pressures lead to the reported increased risk of infections in non-heterosexual communities. Evidence indicates that LGBTQ+ adults experience disproportionately higher rates of substance use and substance use disorders relative to their heterosexual counterparts. These health disparities may be partially attributed to minority stress theory, first articulated by Meyer [81,83]. This theory proposes that individuals from stigmatized minority groups experience excess stress arising from their marginalized social position. This chronic stress stems from experiences of prejudice, discrimination and internalized stigma within a heteronormative society, ultimately increasing vulnerability to mental health problems and maladaptive coping behaviours such as substance use. One study returned from our search adds to this notion, in that higher rates of methamphetamine use were a predictor for STIs in homosexual men [38,84]. Race/ethnicity was associated with an elevated risk of infection. Across multiple countries, Black and South Asian populations were reported as having a greater risk of infection than the comparative White populations. Black populations were disproportionately more affected by STIs [40,85] than their White counterparts in both the UK and the USA [86,87]. This may indicate the interplay of social settings and healthcare disparities. Within challenging social settings, people of colour, particularly Black communities, have a greater vulnerability to STIs, due to socioeconomic disparities, limited access to quality education and increased prevalence of high-risk sexual behaviours in marginalized communities [86].

Other infectious diseases were also noted upon analysis of race/ethnicity. Argamany reported a greater incidence of *C. difficile* in White populations than in Black populations. However, the latter had significantly higher rates of mortality and severity of *C. difficile* cases. Given that *C. difficile* infection is strongly associated with antibiotic exposure [88,89], one could infer that the observed racial disparities in incidence may reflect differential patterns of antibiotic prescribing across ethnic groups [84,87] or variations in healthcare utilization frequency between racial/ethnic populations [86]. Similar studies also highlight that the rate of *C. difficile* increased with greater levels of income, which demonstrates the complex interplay between ethnicity and socioeconomic deprivation, and their respective relationships with infection risk. Future work needs to separate the effects of ethnicity and social deprivation as people from ethnic minorities are more likely to live in deprived areas.

Age was also associated with an elevated risk of infection. Extremes of age ranges such as the elderly and infants were reported to be at higher risk of infectious diseases such as septicaemia, tuberculosis and *C. difficile*. The elderly are known to suffer from multi-morbidities which may increase their susceptibility to infection [90,91]; conversely, infants are vulnerable to infection due to developing immune systems [92]. STIs were most prevalent in young adults, which is likely explained by behavioural differences between this substratum of the population compared to other age groups.

The relationship between sex and infection risk was varied. In adult populations, female sex was associated with an elevated risk of STIs, with anatomical differences likely to contribute to these inequalities in infection risk [93,95]. The evidence base details that men are at greater risk of contracting infectious diseases such as TB, with some risk factors, such as smoking, alcohol consumption, poor nutrition and human immunodeficiency virus comorbidity being noted [93,94]. The differing risks for females and males regarding STIs and infectious diseases are multifaceted and influenced by a blend of biological, physiological, behavioural and social factors [95,98].

Pregnancy and maternity were also associated with an elevated risk of infection, with increased risk of STIs reported in pregnant women [57]. However, in studies that reported higher prevalence of STIs in pregnant women, this was substantially more so in women of younger age, non-Hispanic Black race/ethnicity and women who were unmarried [57]. This could indicate that whilst pregnancy itself makes women more susceptible to infection due to physiological changes [99], the reasoning for the elevated risk of STIs is multifaceted, due to age, ethnicity and income, all of which are factors that we have explored in this scoping review.

### Future work

The lack of published surveillance reports identified as part of the grey literature searches suggests a gap in nations’ routine surveillance of bacterial infections amongst populations with factors associated with health inequalities. Strengthening routine surveillance reporting to include data on health inequalities may help draw attention to any evident disparities in infection burden amongst these populations. One example of this is the inclusion of a dedicated health inequalities outcome within the 2024–29 National Action Plan for AMR [100] following on from the enhanced reporting of health inequalities data within the annual English Surveillance Programme for Antimicrobial Utilisation and Resistance report [101,102]. Furthermore, future work could involve assessing the overlap between bacterial and viral infection and identifying the extent to which types of infection are related to different health inequalities.

### Strengths and limitations

The review demonstrates notable strengths by adherence to a systematic methodology in line with PRISMA guidelines, enhancing credibility and ensuring a high standard of research practices.

In terms of limitations, this study only considered the impact of factors associated with health inequalities on the risk of bacterial infections. Many reviews have been conducted on viral infections such as COVID-19, which notably highlighted significant risk factors for COVID-19 mortality. Evidence reports that the rates of infection and mortality were higher in older people, less-abled individuals, those from deprived or poverty-stricken backgrounds, those of non-White ethnic backgrounds and those with learning disabilities [103,107]. In large part, these observations were reportedly compounded due to pre-existing health inequalities.

Unsurprisingly, most of the research in this area focused on STIs and tuberculosis, infections commonly associated with health inclusion groups. There is a clear gap in the literature for the association of health inequalities and the risk of bacterial infection with priority pathogens such as *Acinetobacter baumannii*, *Pseudomonas aeruginosa* and *Enterobacteriaceae* [108].

## Conclusions

This scoping review demonstrated emerging evidence that highlights an association between elements of health inequalities and the heightened risk of bacterial infections within high-income countries. Published literature evidence available up to 2021 highlighted that this was most notable for ethnic populations, members of the LGBTQ+ community, people experiencing homelessness and those of a poor socioeconomic status, showing that these populations are at a higher risk of bacterial infection. Furthermore, grey literature searches found no publications of surveillance reports containing data on health inequalities in the burden of bacterial infections. Improved national routine surveillance and subsequent targeted interventions may be beneficial in terms of identifying and addressing modifiable risk factors that are driving increased risks of infection.

## Supplementary material

10.1099/acmi.0.000956.v4 Supplementary Material 1.

## Abbreviations

AMR: antimicrobial resistance
PRISMA: Preferred Reporting Items for Systematic Reviews and Meta-Analyses
STEC: Shiga toxin-producing *Escherichia coli*
STI: sexually transmitted infection
TB: *Mycobacterium tuberculosis*
